# Immunometabolic dysregulation in autoimmune rheumatic diseases: the central role of glycolytic reprogramming in pathogenesis and traditional Chinese medicine therapy

**DOI:** 10.3389/fimmu.2026.1754832

**Published:** 2026-03-30

**Authors:** Jianting Wen, Jian Liu, Lei Wan, Fanfan Wang, Yang Li

**Affiliations:** 1 Department of Rheumatology and Immunology, First Affiliated Hospital of Anhui University of Chinese Medicine, Hefei, Anhui, China; 2 Institute of Rheumatology, Anhui Academy of Chinese Medicine, Hefei, Anhui, China; 3 Anhui Province Key Laboratory of Modern Chinese Medicine Department of Internal Medicine Application Foundation Research and Development, Hefei, Anhui, China

**Keywords:** autoimmune rheumatic diseases, glycolytic reprogramming, immunometabolic dysregulation, rheumatoid arthritis, traditional Chinese medicine

## Abstract

Immunometabolic dysregulation has emerged as a key driver in the pathogenesis of autoimmune rheumatic diseases (ARDs), including rheumatoid arthritis (RA), osteoarthritis (OA), and systemic lupus erythematosus (SLE). This review highlighted the central role of glycolytic reprogramming in driving immune cell dysfunction and disease progression. In RA, enhanced glycolysis promoted T cell dysregulation, synovial fibroblast activation, and macrophage polarization. In OA, glycolytic alterations in chondrocytes and synovial tissues were central to disease pathology, while SLE was characterized by metabolic shifts in podocytes, T cells, and NETosis processes. Traditional Chinese medicine (TCM) may be a promising therapeutic strategy by targeting glycolytic pathways to modulate immune responses and restore metabolic balance. Despite existing challenges, the integration of multi-omics and artificial intelligence (AI) may facilitate the development of personalized immunometabolic therapies. This review underscored glycolysis as a pivotal therapeutic target and advocated for interdisciplinary approaches in future ARD research.

## 1 Introduction

Autoimmune rheumatic diseases (ARDs) [including rheumatoid arthritis (RA), systemic lupus erythematosus (SLE), and osteoarthritis (OA)] are chronic inflammatory disorders characterized by dysregulated immune activation and progressive tissue destruction (1, 2). Although these diseases differ in clinical manifestations, they share the common feature of sustained immune-mediated inflammation and tissue remodeling, which contribute to substantial morbidity and long-term disability (3–6). The precise etiology of ARDs involves a complex interplay between genetic susceptibility and environmental triggers (7). Recent advances indicate that, beyond canonical immune signaling abnormalities, metabolic reprogramming has emerged as a fundamental driver sustaining chronic inflammation and immune dysfunction in these disorders (8–11).

Immunometabolism has revealed that metabolic pathways are not merely passive suppliers of bioenergetic substrates but active regulators of immune cell activation, differentiation, and effector function (12). In ARDs, immunometabolic dysregulation encompasses abnormalities in glucose metabolism, lipid metabolism, and glutamine utilization (13). Among these pathways, the reprogramming of glucose metabolism-particularly the shift toward aerobic glycolysis, commonly referred to as the “Warburg effect”-has emerged as a pivotal mechanism (14). Aerobic glycolysis is characterized by the preferential conversion of glucose to lactate even in the presence of sufficient oxygen, enabling rapid ATP production and the generation of biosynthetic intermediates (15). In ARDs, this glycolytic shift is observed not only in activated immune cells such as T cells, B cells, and macrophages, but also in tissue-resident cells including fibroblast-like synoviocytes (FLS) and podocytes (16, 17).

Importantly, glycolysis is prioritized in this review over other metabolic pathways [such as oxidative phosphorylation (OXPHOS), fatty acid oxidation (FAO), and glutaminolysis] for three major reasons. First, enhanced glycolytic flux has been consistently observed across multiple immune and stromal cell populations in RA, OA, and SLE (14, 16, 17). Second, key glycolytic enzymes (including PKM2, PFKFB3, HK2, and LDHA) exhibit non-metabolic regulatory functions that directly influence cytokine production, Th17 differentiation, inflammasome activation, and epigenetic modifications, thereby mechanistically linking metabolic flux to inflammatory signaling (14, 15). Third, glycolysis represents a therapeutically tractable pathway, with pharmacological inhibitors and metabolic modulators demonstrating efficacy in preclinical models of ARDs (18, 19). These features establish glycolysis as a central and targetable immunometabolic hub rather than a mere secondary metabolic consequence of inflammation.

Currently, there is no curative therapy for ARDs (20). Conventional treatments-including non-steroidal anti-inflammatory drugs (NSAIDs), disease-modifying antirheumatic drugs (DMARDs), and biologics-primarily aim to suppress immune activation without directly addressing the underlying metabolic dysregulation (18). Although these therapies have improved disease management to a certain extent, they are often limited by incomplete response rates, high cost, and potential adverse effects (19).

In this context, metabolic intervention has emerged as a promising complementary therapeutic strategy. Traditional Chinese Medicine (TCM), characterized by its multi-component and multi-target properties, has been increasingly shown to modulate key glycolytic regulators [such as AMPK, mTOR, hypoxic-inducible factor-1α (HIF-1α), and PKM2] (21–24). Rather than acting solely through broad immunosuppression, several TCM-derived compounds have been reported to reprogram immune cell metabolism and restore the glycolysis imbalance associated with chronic inflammation (25–27).

**Figure 1:**
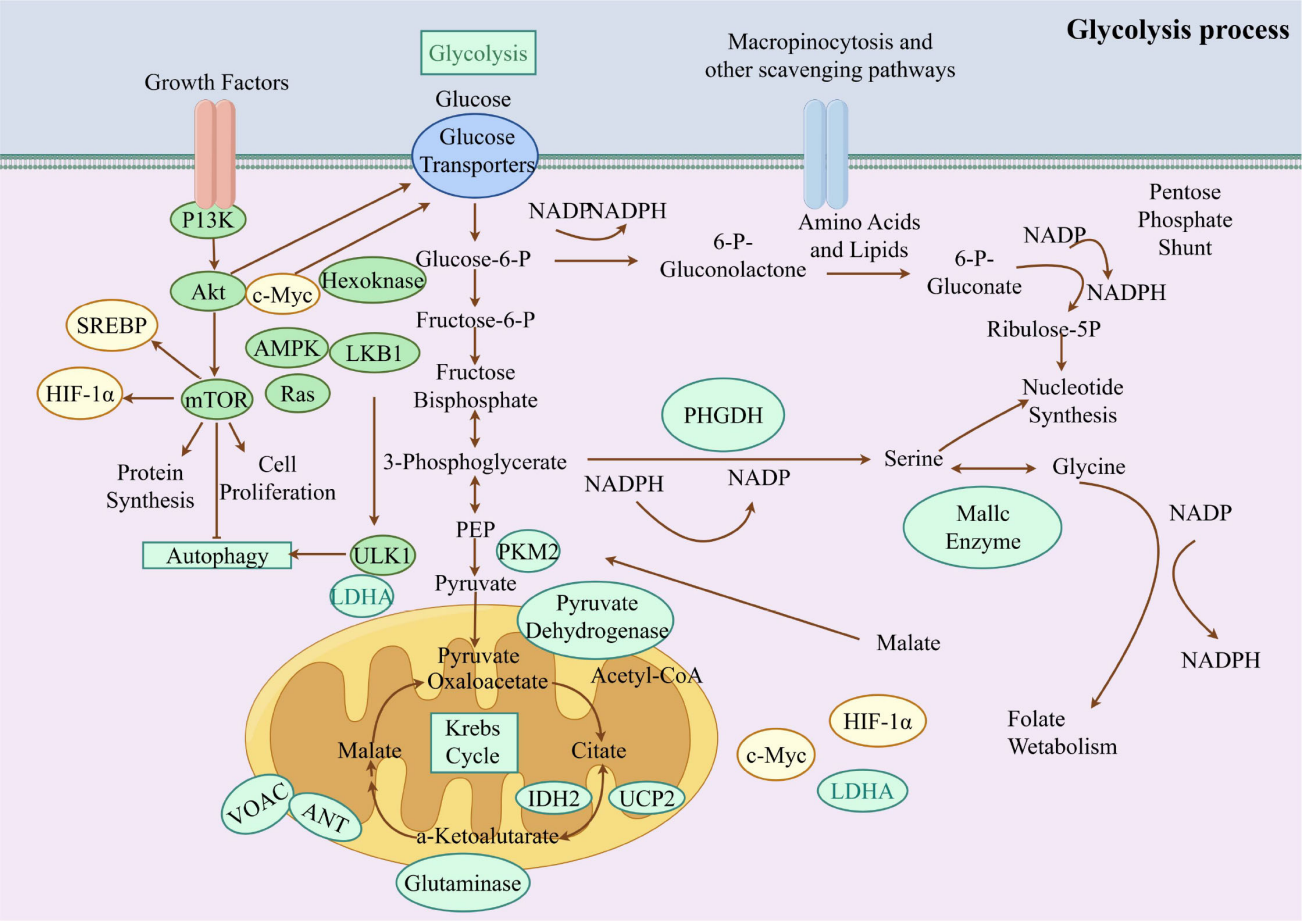
Schematic overview of glycolytic reprogramming.

Therefore, this review synthesized current evidence supporting glycolytic reprogramming as a unifying pathogenic mechanism across RA, OA, and SLE, and critically evaluated how TCM-based interventions intersect with contemporary immunometabolic frameworks see in **Figure 1**. By emphasizing mechanistic integration and therapeutic tractability, the present study aimed to reposition glycolysis as a central immunometabolic axis in the pathophysiology of ARDs.

## 2 Glycolytic reprogramming in RA

RA is a chronic systemic autoimmune disease characterized by persistent synovial inflammation, synovial hyperplasia, and progressive destruction of cartilage and bone (28, 29). It affects approximately 0.5-1% of the global population and shows a higher prevalence in women (30). Its pathogenesis involves a complex interplay among genetic predisposition, environmental triggers, autoantibody production, immune cell infiltration, and sustained release of pro-inflammatory cytokines (31). Accumulating evidence indicates that glycolytic reprogramming represents a unifying metabolic feature across immune and stromal compartments in RA, functioning as a central driver of inflammatory amplification and tissue damage rather than merely a secondary metabolic adaptation (32–34).

### 2.1 T cells and glycolysis

**Figure 2:**
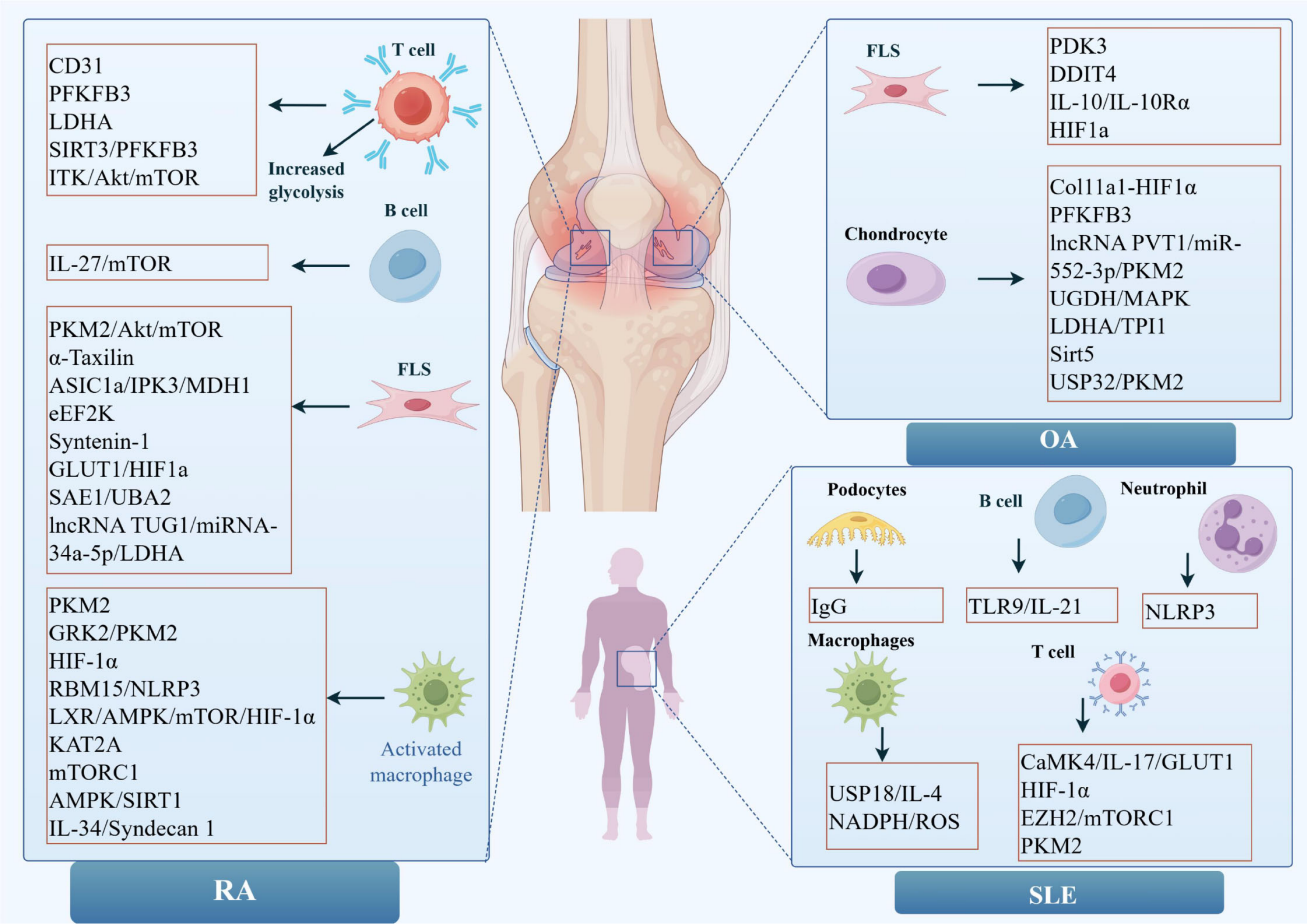
Key regulators and mechanistic networks of glycolytic reprogramming in RADs.

T cell dysfunction in RA is closely associated with enhanced glycolytic reprogramming see in **Figure 2** and **Table 1**. Liu et al. have demonstrated that CD31 ITIMs orchestrate a metabolic switch in regulatory T cells (Tregs), where distinct tyrosine residues regulate fructose utilization or mitochondrial function via PFKFB3 and RNF111/OGT, respectively (35). Transcriptional network analyses have further identified PFKFB3 and GAPDH as central metabolic nodes in CD8^+^ T cells derived from RA patients (36, 37). Elevated LDHA activity sustains aerobic glycolysis, promoting pro-inflammatory effector functions and hypoxia-adapted proliferation of CD8^+^ T cells (38). Upstream regulatory inputs further reinforce this glycolytic dependency. SIRT3 deficiency downregulates PFKFB3, impairs ATP production, and exacerbates arthritic severity (39). Additionally, T cell-derived TNF-α activates the ITK-Akt-mTOR axis, promoting glycolysis, mitochondrial biogenesis, and the differentiation of pro-inflammatory CD4^+^ T cell subsets (40). Collectively, these findings suggest that glycolysis in RA T cells operates as a signaling-integrated metabolic program, linking mTOR activation, enzyme regulation, and inflammatory differentiation into a feed-forward immunometabolic loop.

**Table 1:** Key regulators and mechanisms of glycolytic reprogramming in RA.

Models	Therapeutic tractability	Key regulators	Mechanisms
CIA	Diagnostic (clinical)	Hexokinase and fructose-bisphosphate aldolase	As new markers for the diagnosis of RA
RA patients	Diagnostic (clinical); Small molecule (animal)	PKM2	As a clinically useful indicator for evaluating disease activity and RA diagnosis
Treg	Genetic (*in vitro*)	CD31	CD31 Y686F mutation disrupts glycolysis in Tregs, shifting energy production towards mitochondrial function via the RNF111/OGT pathway.
CD8^+^ T cells	Genetic (*in vitro*); Small molecule (animal)	PFKFB3	PFKFB3 and MYC in CD8+ Tem and Temra cells as a key pathological feature in RA T lymphocytes
CD8^+^ T cells	Small molecule (*in vitro*)	LDHA	Increased LDHA activity and aerobic glycolysis can reduce the deleterious inflammatory in RA CD8^+^ T cells
AIA CD4^+^ T cells	Genetic (animal); Small molecule (animal)	SIRT3/PFKFB3	SIRT3 deficiency reduces PFKFB3-driven T-cell glycolysis and promotes RA inflammation
CD4^+^ T cells	Small molecule (clinical); Biologic (clinical)	ITK/Akt/mTOR	ITK-Akt-mTOR to drive CD4^+^ T cell metabolic reprogramming, which is dysregulated in RA
CD4^+^ T cells	Small molecule (*in vitro*)	PFKFB3	Survivin promotes a glycolytic switch in CD4^+^ T cells by suppressing the transcription of PFKFB3 in RA
B cell	Biologic (clinical); Small molecule (animal)	IL-27/mTOR	Enhanced cellular glycolysis of RA B cells induced by IL-27 may contribute to B cells hyperactivation through activating the mTOR signaling pathway
RA-FLS	Small molecule (animal)	PKM2/Akt/mTOR	PKM2 mediates glycolytic reprogramming to induce the release of RA-FLSs inflammatory cytokines by activating the Akt/mTOR signaling pathway, thereby promoting the progression of RA
RA-FLS	Genetic (*in vitro*)	α-Taxilin	Alpha-taxilin interacts with key glycolytic enzymes associated with metabolic shifts in FLS, which as a potential diagnosis and therapeutics target in RA
RA-FLS	Small molecule (animal)	ASIC1a/IPK3/MDH1	RIPK3 promotes ASIC1a-mediated FLS migration and invasion via malate shuttle-driven mitochondrial respiration in RA
RA-FLS	Small molecule (animal)	eEF2K	eEF2K inhibition suppressed glycolysis and aggressive behaviors of RA-FLS
RA-FLS and endothelial cell	Genetic (*in vitro*)	Syntenin-1	Metabolic reprogramming by Syntenin-1 directs RA-FLS and endothelial cell-mediated inflammation and angiogenesis
RA-FLS	Small molecule (animal)	GLUT1/HIF1a	TNF induces glycolytic reprogramming of FLS and the potency of immunometabolism for RA
RA-FLS	Genetic (*in vitro*)	SAE1/UBA2	SAE1/UBA2 may contribute to synovial glycolysis and joint inflammation in RA
RA-FLS	RNA-based (*in vitro*); Small molecule (animal)	lncRNA TUG1/miRNA-34a-5p/LDHA	LncRNA TUG1-mediated glucose metabolism and apoptosis of FLSs-RA through modulating the miR-34a-5p-LDHA pathway

### 2.2 B cells and glycolysis

B cells contribute to RA pathogenesis primarily through the production of autoantibodies (such as RF and anti-CCP) (41). Enhanced glycolysis supports B cell activation and differentiation. IL-27 has been shown to promote glycolysis in peripheral B cells via the mTOR signaling, thereby driving proliferation and inflammatory responses (42). Inhibition of glycolysis with 2-deoxy-D-glucose or blockade of the mTOR pathway attenuates these effects, highlighting glycolysis as a critical metabolic checkpoint in B cell-mediated autoimmunity. These data indicate that glycolytic flux not only sustains B cell bioenergetic demands but also modulates signaling pathways essential for autoantibody production.

### 2.3 Macrophages and glycolysis

Macrophages are central mediators of synovial inflammation in RA (43). Their pro-inflammatory activation is tightly coupled to enhanced glycolytic metabolism. Upregulation of PKM2 promotes the production of TNF-α and IL-1β via STAT1 signaling (44). Loss of GRK2-mediated phosphorylation and de-succinylation of PKM2 amplifies glycolytic flux and inflammatory output (45). The glycolysis-HIF-1α axis forms a pathogenic feedback loop, in which RA serum induces IL-1β production through enhanced glycolysis (46). Conversely, RBM15 reduces macrophage glycolysis and suppresses NLRP3 inflammasome activation, revealing an epigenetic layer of metabolic regulation (47). Pharmacological targeting has shown therapeutic potential. The LXR inverse agonist SR9243 attenuates arthritis by inhibiting glycolysis via the AMPK/mTOR/HIF-1α signaling (48). Additional regulators include KAT2A, which promotes NLRP3 activation via glycolytic enhancement (49); Zip8-mediated zinc influx, which activates mTORC1-driven glycolysis (50); and AMPK/SIRT1 deficiency, which enhances pro-inflammatory polarization (51). Notably, 2-DG shifts macrophages toward an M2 phenotype in an AMPK-dependent manner (52). Furthermore, IL-34 drives glycolytic M34 macrophage differentiation, thereby promoting osteoclastogenesis and inflammation (53). These studies collectively demonstrate that glycolysis functions as a metabolic switch controlling macrophage polarization and inflammasome activation in RA.

### 2.4 FLS and glycolysis

FLS are key effector cells in RA (54). Their pathogenic phenotype is sustained by enhanced glycolytic metabolism (55). PKM2 upregulation drives lactate production and cytokine release via the Akt/mTOR signaling (56). α-Taxilin interacts with glycolytic enzymes, contributing to metabolic dysregulation (57). The acidic microenvironments activate the ASIC1a-RIPK3 axis, enhancing mitochondrial respiration supported by glycolytic flux (58). eEF2K promotes glycolysis and FLS invasion (59). Syntenin-1, via SDC-1, enhances glycolytic activation through mTOR and HIF-1α signaling (60). TNF stimulates glycolysis through the TAK1/HIF1A/GLUT1 pathway (61). Post-translational regulation further fine-tunes glycolysis: SAE1/UBA2-mediated SUMOylation promotes PKM2 nuclear translocation (62), while lncRNA TUG1 enhances LDHA expression by sponging miR-34a-5p (63). Thus, glycolytic reprogramming in RA-FLS is controlled at transcriptional, post-translational, and non-coding RNA levels, collectively reinforcing their invasive and inflammatory phenotype.

### 2.5 Integrated glycolytic regulatory network in RA

Although T cells, B cells, macrophages, and FLS are often studied in isolation, emerging evidence indicates that glycolytic reprogramming constitutes a metabolically interconnected network within the rheumatoid synovium. Shared regulatory nodes (including HIF-1α, PKM2, PFKFB3, mTOR signaling, and AMPK pathways), coordinate inflammatory responses with metabolic adaptation (14–17). Enhanced lactate production further shapes the local microenvironment, reinforcing hypoxia signaling and sustaining immune activation. Rather than acting as a passive metabolic consequence of inflammation, glycolysis in RA functions as a feed-forward amplifier that integrates cytokine signaling, epigenetic modification, and cellular differentiation. This integrated framework positions glycolysis as a central and therapeutically tractable immunometabolic axis in RA.

## 3 Glycolytic reprogramming in OA

OA is a prevalent degenerative joint disease characterized by progressive cartilage degradation, synovitis, and subchondral bone remodeling (64). Its incidence increases with aging and obesity, posing a substantial public health burden (65). Although traditionally regarded as a mechanically driven degenerative disorder, accumulating evidence suggests that metabolic reprogramming-particularly altered glycolysis-contributes to inflammatory amplification and disruption of cartilage homeostasis in OA (66).

### 3.1 Macrophages and glycolysis

Synovial macrophages play a key role in OA-associated inflammation and cartilage damage, see in **Table 2**. IL-10Rα deficiency promotes glycolysis via HIF-1α activation, thereby linking macrophage metabolic state to chondrocyte ferroptosis (67). Additionally, inflammatory FLS-derived exosomes enhance macrophage glycolysis and M1 polarization in a HIF1A-dependent manner, aggravating OA progression (68). Conversely, inhibition of glycolysis with 2-DG or targeting of HIF1A alleviates macrophage-driven inflammation and cartilage degeneration. Collectively, these findings indicate that macrophage glycolytic activation is not merely a correlate of inflammation but an active driver of synovitis and cartilage deterioration in OA.

**Table 2:** Key regulators and mechanisms of glycolytic reprogramming in OA.

Models	Therapeutic tractability	Key regulators	Mechanisms
FLS	Genetic (*in vitro*); Small molecule (animal)	DDIT4	DDIT4 mitigates high glucose-induced FLS activation and OA progression by suppressing glycolysis
FLS	Biologic (animal)	IL-10/IL-10Rα	IL-10Rα deficiency exacerbates OA by promoting HIF-1α-driven macrophage glycolysis and chondrocyte ferroptosis
FLS	Small molecule (animal)	HIF1a	Inflammatory fibroblast-derived exosomes promote OA by driving HIF1A-dependent glycolysis and M1 macrophage polarization
Chondrocyte	Genetic (*in vitro*)	PKM2	PKM2 promotes chondrocyte senescence in OA through a metabolic shift, but does not regulate inflammatory responses
Chondrocyte	Genetic (animal)	Col11a1-HIF1α	COL11A1 loss disrupts chondrocyte homeostasis via a HIF1α-mediated glycolysis-OXPHOS shift in OA
Chondrocyte	Small molecule (animal)	PFKFB3	PFKFB3 alleviates OA by enhancing glycolysis and suppressing ER stress-induced chondrocyte apoptosis
Chondrocyte	RNA-based (*in vitro*)	lncRNA PVT1/miR-552-3p/PKM2	LncRNA PVT1 upregulation exacerbates OA progression via the miR-552-3p/PKM2 axis to promote glycolysis and chondrocyte damage
Chondrocyte	Small molecule (*in vitro*)	UGDH/MAPK	Lactate promotes OA progression by inducing UGDH lactylation, which activates MAPK signaling and exacerbates extracellular matrix degradation
Chondrocyte	Small molecule (animal)	LDHA/TPI1	LDHA promotes OA progression via H3K18 lactylation-driven TPI1 transcription and enhanced glycolysis
Chondrocyte	Genetic (animal)	Sirt5	Sirt5 deficiency exacerbates OA by disrupting chondrocyte metabolism via elevated malonylation
Chondrocyte	Genetic (animal)	USP32/PKM2	USP32 promotes TMJ OA by deubiquitinating and stabilizing PKM2 to enhance glycolysis and chondrocyte dysfunction
Mesenchymal stem cells-Th17 cells	Genetic (animal)	PIEZO1	Mechanical activation of Piezo1 in MSCs promotes OA via glycolysis-dependent crosstalk with Th17 cells

### 3.2 Synovial stromal cells and glycolysis

FLS in OA undergo metabolic reprogramming similar to that observed in RA. PDK3 overexpression drives glycolytic shifts in proliferative THY1^+^ FLS, whereas PDK inhibition redirects metabolism toward oxidative phosphorylation and reduces inflammation (69). Downregulation of DDIT4 in diabetic OA enhances glycolysis through upregulation of HK2 and PKM2, thereby aggravating cartilage injury (70). These studies highlight glycolytic remodeling in synovial stromal cells as a key mediator of inflammatory-metabolic crosstalk in OA joints.

### 3.3 Chondrocytes and glycolysis

Chondrocytes represent the principal structural cells in cartilage and exhibit extensive glycolytic reprogramming in OA. Chondrocyte senescence is a central driver in OA pathogenesis, particularly in aging populations, where it promotes extracellular matrix degradation (71). PKM2 regulates chondrocyte senescence independently of inflammatory signaling (72). Silencing PKM2 reduces senescence markers (such as p16^INK4a and SA-β-Gal), while enhancing collagen type II expression. COL11A1 deficiency disrupts the glycolysis-OXPHOS balance via HIF-1α, exacerbating senescence and joint degeneration (73, 74). Downregulation of PFKFB3 impairs glycolysis, ATP production, and lactate output, thereby promoting ER stress and apoptosis (75). Conversely, restoration of PFKFB3 enhances glycolysis and mitigates PERK and CHOP signaling. LncRNA PVT1 promotes PKM2 expression by sponging miR-552-3p, leading to enhanced glycolysis and apoptosis (76). Elevated lactate promotes lactylation of UGDH at K6, suppressing glycosaminoglycan synthesis and activating the MAPK signaling (77, 78). LDHA-mediated H3K18 lactylation enhances TPI1 transcription and glycolysis (79). Sirt5 deficiency increases lysine malonylation, impairing glycolytic carbon metabolism (80). USP32 stabilizes PKM2 via deubiquitination, enhancing glycolysis and contributing to mitochondrial dysfunction in TMJOA (81). Collectively, these findings demonstrate that glycolytic reprogramming in chondrocytes is regulated at enzymatic, epigenetic, and post-translational levels, directly linking metabolic imbalance to cartilage degeneration.

### 3.4 T cell-mesenchymal stem cells metabolic crosstalk

Mechanical stimulation activates Piezo1 in MSCs, increasing HK2-driven glycolysis and promoting Th17 polarization through macrophage MIF signaling (82). This evidence suggests that glycolysis mediates mechano-inflammatory signaling networks in OA, extending metabolic control beyond individual cell types.

### 3.5 Integrated glycolytic regulatory network in OA

Across macrophages, synovial stromal cells, chondrocytes, and MSCs, glycolytic reprogramming converges on shared regulators, including HIF-1α, PKM2, PFKFB3, and mTOR signaling (66–70, 83, 84). Lactate accumulation further modulates post-translational modifications (such as lactylation), establishing a metabolic and epigenetic feedback loop. Thus, glycolysis in OA should be understood not solely as a degenerative metabolic consequence but as an active regulator that integrates inflammatory, mechanical, and epigenetic signals within the joint microenvironment.

## 4 Glycolytic reprogramming in SLE

SLE is a multi-organ autoimmune disease characterized by loss of self-tolerance and immune complex-mediated tissue damage (85). Beyond canonical immune dysregulation, enhanced glycolytic metabolism has emerged as a defining feature of pathogenic immune subsets in SLE (86–88).

### 4.1 T cells and glycolysis

CD4^+^ T cells from patients with SLE exhibit elevated glycolytic capacity that correlates with disease activity (89). CaMK4 inhibition reduces glucose-6-phosphate and pyruvate levels, downregulates GLUT1 expression, and suppresses Th17 differentiation (90). Elevated HIF-1α expression correlates with glycolysis-associated genes and enhances Th17 differentiation (91, 92). EZH2 overexpression in lupus CD4^+^ T cells is driven by glycolysis and mTORC1 activation, linking metabolic flux to epigenetic reprogramming. PKM2 promotes Tfh differentiation, whereas activation of TEPP-46 suppresses PKM2 nuclear translocation and ameliorates disease manifestations. These findings underscore glycolysis as a metabolic-epigenetic axis that shapes pathogenic T cell subsets in SLE see in **Table 3**.

**Table 3:** Key regulators and mechanisms of glycolytic reprogramming in SLE.

Models	Therapeutic tractability	Key regulators	Mechanisms
Podocytes		IgG	Abnormal IgG glycosylation in lupus nephritis induces podocyte injury through glycolytic suppression, reversible upon treatment
B cells		TLR9/IL-21	TLR9 and IL-21 synergistically promote plasmocyte differentiation in SLE by enhancing glycolysis to upregulate IL-21 receptor
Neutrophil		NLRP3	NETs promote placental NLRP3 lactylation and pyroptosis via glycolysis in lupus pregnancies
Macrophages		USP18/IL-4	USP18 attenuates M1 macrophage polarization in SLE by suppressing glycolysis and mitochondrial activity
Macrophages		NADPH/ROS	ALD-DNA drives macrophage inflammation in lupus by reprogramming glucose metabolism to elevate ROS via suppressed PPP
T cell		CaMK4/IL-17/GLUT1	CaMK4 promotes glycolysis to drive IL-17 production and exacerbate SLE activity
CD4^+^ T cells		HIF-1α	HIF-1α upregulation in SLE CD4^+^ T cells promotes glycolysis and Th17 differentiation, correlating with SLE disease activity
CD4^+^ T cells		EZH2/mTORC1	mTORC1 and glycolysis upregulate EZH2 in lupus CD4^+^ T cells by suppressing miR-26a and miR-101
T cell		PKM2	PKM2 tetramerization attenuates SLE by inhibiting glycolysis and follicular helper T-cell differentiation

### 4.2 B cells and glycolysis

B cells play a pivotal role in SLE pathogenesis by producing autoantibodies that drive autoimmune responses. Emerging evidence highlights that metabolic reprogramming, particularly enhanced glycolysis, is crucial for supporting the hyperactivation and functional demands of B cells in this disease. As demonstrated by Kim et al., cTfh17 cells from SLE patients exhibit heightened glycolytic activity, which is critical for their capacity to activate B cells (93). Glucose deprivation or glycolysis inhibition significantly impairs the expression of costimulatory molecules and cytokine production in cTfh17 cells, thereby reducing their ability to promote B cell responses. This finding underscores a key metabolic dependency in T cell-driven B cell hyperactivity in SLE. Furthermore, intrinsic metabolic changes within B cells themselves are also glycolysis-dependent. In SLE, TLR9 stimulation rapidly enhances glycolysis, leading to dissociation of GAPDH from IL-21R mRNA and subsequent upregulation of IL-21 receptor expression. Inhibition of glycolytic enzymes HK2 and GAPDH blocks this process, indicating that glycolytic metabolism is essential for amplifying B cell receptor signaling and promoting plasmocyte differentiation in lupus (94). Therefore, both extrinsic T cell help and intrinsic B cell activation in SLE are critically supported by glycolytic metabolism. Targeting these metabolic pathways may offer novel therapeutic strategies to modulate B-cell hyperactivity and ameliorate SLE progression.

### 4.3 Macrophages and glycolysis

USP18 deficiency enhances macrophage M1 polarization by promoting glycolysis and HIF-1α expression (95). ALD-DNA reprograms macrophage metabolism toward glycolysis while suppressing the pentose phosphate pathway, leading to increased ROS production (96). Together, these studies demonstrate that macrophage glycolytic reprogramming amplifies inflammatory cascades in SLE.

### 4.4 Tissue-resident cells (podocytes) and glycolysis

Aberrant IgG glycosylation impairs podocyte glycolysis in lupus nephritis, altering pyruvate metabolism and reducing glycolytic rates (88). This finding highlights that glycolytic dysregulation in SLE extends beyond immune cells to include organ-specific parenchymal cells.

### 4.5 NETosis and glycolytic lactylation

SLE-derived neutrophil extracellular traps (NETs) promote glycolysis-dependent NLRP3 lactylation and trophoblast pyroptosis (97). DNase I or PAD4 deficiency mitigates these effects, underscoring the pathogenic role of NET-associated glycolytic metabolites. This illustrates how glycolytic metabolites (such as lactate) can modulate inflammasome signaling via epigenetic modification.

### 4.6 Integrated glycolytic regulatory network in SLE

Across T cells, B cells, macrophages, podocytes, and NETs, glycolytic reprogramming converges on shared hubs (including HIF-1α, PKM2, mTORC1, and lactate-mediated epigenetic modifications) (86–97). Enhanced glycolysis not only meets bioenergetic demands but also orchestrates transcriptional programs, inflammasome activation, and subset differentiation. Therefore, glycolysis in SLE functions as a systemic immunometabolic driver, integrating adaptive and innate immune dysregulation with organ-specific injury.

## 5 Therapeutic interventions targeting glycolysis by TCM in ARDs

**Figure 3:**
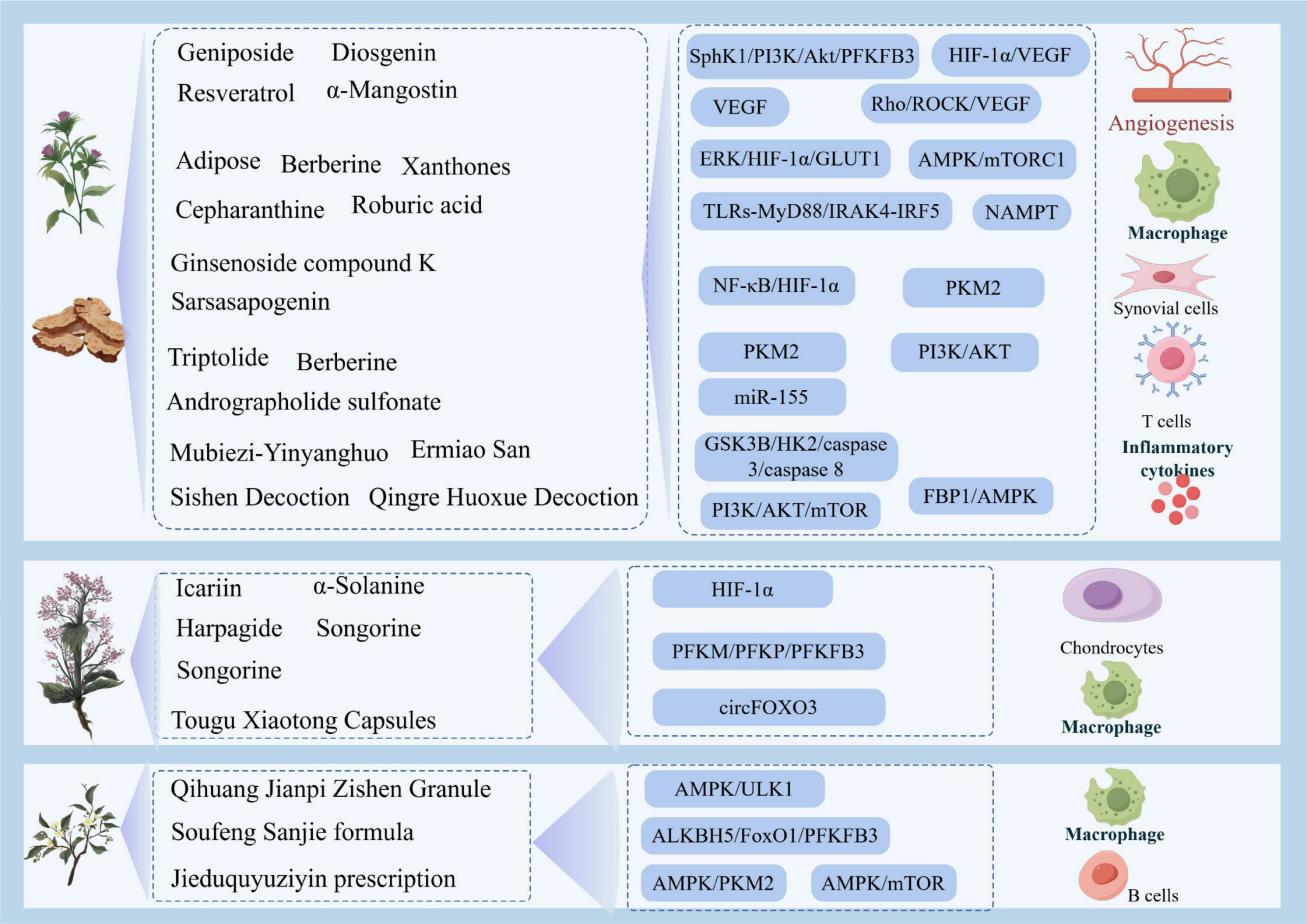
Therapeutic mechanisms of TCM targeting glycolytic pathways in ARDs.

Targeting glycolysis has emerged as a promising therapeutic strategy in ARDs, given its pivotal role in activating immune cells and perpetuating inflammation. An increasing body of evidence underscores the potential of TCM in modulating this metabolic pathway. Through their characteristic multi-component and multi-target mechanisms, various TCM compounds and active compounds have been shown to suppress aberrant glycolytic flux in pathogenic immune cells. Consequently, TCM-mediated glycolytic modulation restores immune metabolism balance, promotes immune homeostasis, and attenuates disease progression in preclinical models of ARDs see in **Figure 3** and **Table 4**. These findings position TCM as a promising source of novel metabolic immunomodulators, offering a compelling alternative or adjunctive strategy to complement conventional therapies for ARDs.

**Table 4:** TCM interventions targeting glycolytic pathways in ARDs.

RADs	Models	Key regulators	TCM	Mechanisms
RA	AA rat models and HUVECs	SphK1/PI3K/Akt/PFKFB3	Geniposide	Geniposide suppresses RA angiogenesis by inhibiting the SphK1-PI3K-Akt-PFKFB3 signaling axis and glycolysis
	HUVEC	VEGF	Dioscorea spongiosa	Diosgenin, the active component of Bixie, suppresses angiogenesis by inhibiting ROCK1 and downregulating glycolysis in RA
	HUVEC	Rho/ROCK/VEGF	RSV	RSV suppresses RA angiogenesis by inhibiting glycolysis-fueled Rho/ROCK/VEGF signaling
	AIA rats and HUVEC	HIF-1α/VEGF	α-MAN	α-MAN alleviates RA by inhibiting glycolysis and subsequent HIF-1α/VEGF-driven angiogenesis
	CIA rats and macrophage	ERK/HIF-1α/GLUT1	Adipose	ADSCs-exosome delivered ICA alleviates RA by suppressing glycolysis and promoting M1-to-M2 macrophage polarization
	Rat RA model and macrophage	ERK/HIF-1α/GLUT1	RBA	RBA-loaded dual-targeting nanoparticles alleviate RA by suppressing glycolysis to drive M1-to-M2 macrophage repolarization
	Macrophage	TLRs-MyD88/IRAK4-IRF5	CEP	CEP alleviates RA by suppressing monocyte chemotaxis and M1 macrophage polarization via glycolytic and TLR/IRF5 pathways
	AA rat models and macrophage	AMPK/mTORC1	Berberine	Berberine alleviates RA via AMPK-dependent inhibition of mTORC1/HIF-1α-driven glycolysis in M1 macrophages
	CIA rats and macrophage	NAMPT	Xanthones from Securidaca inappendiculata Hassk	Xanthone-rich fraction alleviates arthritis by inhibiting NAMPT/glycolysis to repolarize macrophages from M1 to M2
	AA rats	NF-κB/HIF-1α	Ginsenoside CK	CK alleviates RA by activating glucocorticoid receptor to inhibit glycolysis and NF-κB/HIF-1α pathway
	AA rats and RA-FLS	PKM2	Sarsasapogenin	Sarsasapogenin exerts anti-arthritic effects by targeting PKM2 to inhibit glycolysis and pathological behaviors of FLS
	CIA rats and Th17 cell	PKM2	TP	TP alleviates arthritis by inhibiting PKM2-mediated glycolysis and Th17 cell differentiation
	AIA rats and Th17 cells	PI3K/AKT	AS	AS ameliorates arthritis by suppressing HK2-mediated glycolysis and PI3K/AKT-driven Th17 differentiation
	AA rats and CD4+T cells	miR-155	Berberine	Berberine alleviates arthritis by suppressing M1-exosomal miR-155 transfer, thereby inhibiting CD4+ T cell glycolysis and restoring Th17/Treg balance
	RA-FLS	GSK3B/HK2/caspase 3/caspase 8	MBZ-YYH herb pair	The MBZ-YYH herb pair combats RA by targeting HK2 and caspases to suppress FLS glycolysis and proliferation
	RA-FLS	PI3K/AKT	ASSD	ASSD alleviates RA via kaempferol/luteolin/quercetin-mediated PI3Kδ/AKT/glycolysis inhibition
	CIA rats	PI3K/AKT/mTOR	EMS	EMS alleviates RA by inhibiting PI3K/AKT/mTOR/HIF-1α-mediated glycolysis in FLS
	CIA mice	FBP1/AMPK	QRHXD	QRHXD ameliorates RA by inhibiting FBP1 and activating AMPK signaling to reduce inflammation
OA	Chondrocytes	HIF-1α	ICA	ICA enhances chondrocyte viability and extracellular matrix synthesis by upregulating HIF-1α and anaerobic glycolysis
	Chondrocytes	HIF-1α	α-Solanine	α-Solanine-loaded nano-delivery system ameliorates OA by suppressing HIF-1α-mediated glycolysis and ferroptosis
	Chondrocytes	PFKM/PFKP/PFKFB3	Harpagide	Harpagide ameliorates OA by binding to glycolytic enzymes and inhibiting TNF-α-induced chondrocyte inflammation
	Chondrocytes	PFKFB3	Songorine	Songorine ameliorates OA by targeting PFKFB3 to suppress glycolysis-driven histone lactylation and inflammatory signaling
	Macrophage	PFKFB3	Songorine	Songorine alleviates OA by targeting PFKFB3 to inhibit glycolysis, histone lactylation, and inflammatory signaling
	Chondrocytes	circFOXO3	TGXTC	TGXTC ameliorate OA by upregulating circFOXO3 to suppress glycolysis in chondrocytes
SLE	Macrophage	AMPK/ULK1	QJZG	QJZG ameliorate lupus nephritis by activating the AMPK/ULK1 pathway to suppress M1 macrophage polarization
	Th17 cells	ALKBH5/FoxO1/PFKFB3	Soufeng Sanjie formula	Soufeng Sanjie formula alleviates lupus and joint injury via the ALKBH5-FoxO1-PFKFB3 axis to enhance glycolysis in monocytic myeloid-derived suppressor cells
	B cell	AMPK/PKM2	JP	JP ameliorates SLE by activating AMPK/PKM2 signaling to inhibit glycolysis-dependent B cell activation
	T cell	AMPK/mTOR	JP	JP alleviates SLE by inhibiting glycolysis and restoring Th17/Treg balance via AMPK/mTOR signaling

### 5.1 TCM treats RA through the glycolysis pathway

#### 5.1.1 TCM monomers

##### 5.1.1.1 TCM inhibits RA angiogenesis through the glycolysis pathway

In RA, pathological angiogenesis and enhanced glycolysis synergistically drive synovial inflammation and joint destruction (98). Emerging evidence reveals that TCM-derived active compounds can ameliorate RA by simultaneously targeting key regulators and glycolytic enzymes, thereby normalizing the metabolic-inflammatory crosstalk that sustains disease progression.

For example, enhanced glycolysis in endothelial cells has been shown to critically drive angiogenesis in RA. Geniposide, an active iridoid glycoside from Gardenia jasminoides, exerts anti-angiogenic effects by suppressing the SphK1-PI3K-Akt signaling axis, thereby downregulating the key glycolytic enzyme PFKFB3 (99). This inhibition reduces glycolytic flux and impairs angiogenesis both *in vitro* and *in vivo*, highlighting a metabolic mechanism for its therapeutic potential in RA. Building on the glycolytic regulation in RA angiogenesis, Dioscorea spongiosa (Bixie) and its active constituent diosgenin exhibit potent anti-angiogenic properties. Mechanistically, diosgenin functions as a direct inhibitor of ROCK1 by stabilizing ROCK1 and suppressing its phosphorylation (100). This action leads to the downregulation of glycolytic pathways, thereby impairing the metabolic activity essential for endothelial cell activation and angiogenesis, revealing another natural product-mediated metabolic intervention for RA.

Resveratrol (RSV) offers a distinct regulatory mechanism via protein deacetylation. As a SIRT1 agonist, RSV activates SIRT1-mediated deacetylation, which inhibits glycolysis and subsequent ATP production in endothelial cells (101). This metabolic reprogramming impairs the Rho/ROCK-VEGF angiogenic axis independently of HIF-1α, revealing a novel acetylation-related pathway for suppressing angiogenesis in RA. Moreover, α-Mangostin (MAN) exemplifies another natural compound targeting glycolysis in RA angiogenesis. MAN alleviates hypoxia and synovial angiogenesis by potently suppressing HIF-1α expression (102). This inhibition disrupts aerobic glycolysis, as evidenced by reduced glycolytic metabolites and modulated LDH activity, thereby decreasing VEGF production and endothelial cell proliferation. Thus, MAN underscores HIF-1α as a pivotal target for metabolic intervention in inflammatory angiogenesis.

##### 5.1.1.2 TCM inhibits RA macrophages through the glycolysis pathway

Icariin (ICA) exhibits significant anti-inflammatory and immunomodulatory effects in RA. A recent study has demonstrated that adipose-derived stem cell exosomes loaded with ICA (ADSCs-EXO-ICA) ameliorate RA by modulating macrophage polarization from the pro-inflammatory M1 toward the anti-inflammatory M2 phenotype. This shift is primarily mediated by the suppression of glycolysis via the ERK/HIF-1α/GLUT1 pathway, as validated by reduced glycolytic activity and cytokine levels in *in vitro* and *in vivo* analyses, highlighting the critical role of energy metabolism in RA pathogenesis (103). Roburic acid (RBA), a natural compound isolated from the traditional herb Gentiana macrophylla Pall., possesses notable anti-inflammatory properties. According to data from Jia et al., a dual-targeted nanoparticle delivery system for roburic acid (RBA-NPs) effectively alleviates RA symptoms by reprogramming pro-inflammatory M1 macrophages toward the anti-inflammatory M2 phenotype (104). This phenotypic switch is mechanistically driven by the downregulation of cellular glycolysis via inhibition of the ERK/HIF-1α/GLUT1 signaling axis, highlighting a crucial link between metabolic reprogramming and the resolution of inflammation in RA.

Cepharanthine (CEP), a natural bisbenzylisoquinoline alkaloid, has demonstrated broad anti-inflammatory and immunomodulatory activities. According to Lu et al., CEP effectively attenuates inflammatory arthritis by blocking M1 macrophage polarization (105). This inhibitory effect is mechanistically linked to the suppression of overactivated glycolytic metabolism, as evidenced by downregulated key glycolytic enzymes and reduced citrate levels, ultimately disrupting the TLRs-MyD88/IRAK4-IRF5 signaling axis in M1-polarizing macrophages. Additionally, berberine (BBR) is a natural isoquinoline alkaloid, exhibiting significant anti-inflammatory and immunomodulatory properties. As has been evidenced previously, BBR ameliorates arthritis by restoring macrophage polarization through the AMPK/mTORC1 pathway (106). This restoration is achieved by modulating glycolytic reprogramming in macrophages, wherein BBR-activated AMPK phosphorylates raptor and TSC2 to inhibit the mTORC1/HIF-1α signaling, thereby effectively suppressing glycolysis in pro-inflammatory M1 macrophages. Furthermore, Securidaca inappendiculata Hassk., a traditional anti-rheumatic herb, also demonstrates potent joint-protective effects in RA. Its therapeutic potential, attributed to the xanthone-rich fraction (XRF), is associated with the production of inflammatory cytokines and macrophage polarization. For example, research has indicated that XRF ameliorates collagen-induced arthritis by inhibiting macrophage M1 polarization (107). This therapeutic effect is mechanistically achieved via suppressing the activity of nicotinamide phosphoribosyltransferase (NAMPT), a key regulator of the glycolytic pathway, thereby negatively regulating synovial glycolysis and promoting an M1-to-M2 phenotypic switch.

##### 5.1.1.3 TCM inhibits RA synovial cell proliferation through the glycolysis pathway

Ginsenoside compound K (CK), an active metabolite derived from ginsenosides, exhibits notable anti-arthritic properties by modulating glucocorticoid receptors to suppress inflammatory responses and cellular metabolic dysregulation in synovial tissues. A previous study has shown that CK effectively inhibits glycolysis in FLS by downregulating the NF-κB/HIF-1α pathway via glucocorticoid receptor activation (108). This suppression downregulates key glycolytic enzymes (including GLUT1, HK2, and PKM2), thereby attenuating energy metabolism and inflammation in RA models. Additionally, sarsasapogenin (SA) is a primary bioactive metabolite of Anemarrhena asphodeloides saponins absorbed into systemic circulation, exerting anti-arthritic effects by targeting pivotal metabolic and cellular processes in RA. For instance, Dai et al. have reported that SA potently suppresses pathological glycolysis in RA-FLS by specifically targeting PKM2 (109). It inhibits PKM2 tetramer activity and its phosphorylation, leading to reduced glucose uptake and glycolytic flux. This metabolic inhibition subsequently attenuates FLS proliferation, invasion, and cytokine release, while promoting apoptosis, highlighting PKM2 as a critical therapeutic target in RA.

##### 5.1.1.4 TCM inhibits RA T cell differentiation through the glycolysis pathway

Triptolide (TP) is a key active compound derived from the herb Tripterygium wilfordii, which possesses potent immunosuppressive and anti-inflammatory properties. These properties make it a promising therapeutic agent for autoimmune diseases (such as RA). As demonstrated, TP effectively suppresses T helper 17 (Th17) cell differentiation-a pivotal process in RA pathogenesis-by directly targeting and inhibiting PKM2-mediated glycolysis (110). This inhibition leads to reduced production of key glycolytic metabolites (pyruvate and lactate), thereby disrupting the metabolic reprogramming essential for Th17 cell differentiation and function, highlighting a novel metabolic mechanism for its anti-arthritic effects. Moreover, andrographolide sulfonate (AS) is a sulfonated derivative of andrographolide, demonstrating significant anti-arthritic efficacy. As evidenced, AS directly targets HK2-the rate-limiting enzyme in glycolysis-to restrict glucose uptake and lactate production (111). This inhibition of glycolytic flux subsequently suppresses the PI3K/AKT signaling pathway, a critical axis for Th17 cell differentiation. Thus, the anti-arthritic effect of AS is attributed to its disruption of this glycolysis-mediated pro-inflammatory T cell responses. Similarly, Cai et al. have noted that BBR alleviates experimental arthritis by suppressing the transfer of exosomal miR-155 from M1 macrophages to CD4^+^ T cells (112). This action disrupts the immunometabolic reprogramming of CD4^+^ T cells, notably by reversing the enhanced glycolysis induced by M1 macrophage-derived exosomes, as indicated by reduced extracellular acidification rate (ECAR) and lactate production. Consequently, BBR restores the balance of T cell differentiation, uncovering a novel exosome-mediated metabolic mechanism underlying its therapeutic effects.

##### 5.1.1.5 TCM compounds target glycolysis in RA

The Mubiezi-Yinyanghuo (MBZ-YYH) herb pair, a TCM formulation, demonstrates anti-RA effects by targeting multiple signaling pathways and cellular processes. As revealed by network pharmacology and molecular docking, the MBZ-YYH herb pair exerts its therapeutic effects against RA by targeting key hubs such as HK2, a critical glycolytic enzyme (113). This action is proposed to inhibit glycolysis and proliferation in RA-FLS, primarily through modulation of the IL-17 and TNF signaling pathways, thereby coordinating a multi-target inhibitory effect on FLS metabolism and inflammation. Moreover, Additive Sishen Decoction (ASSD) is a TCM formula, exerting therapeutic efficacy in RA through its key active ingredients (including kaempferol, luteolin, and quercetin). Ren et al. have demonstrated that these core ingredients exert anti-inflammatory effects by directly targeting and inhibiting PI3Kδ, thereby suppressing the PI3K-AKT signaling pathway (114). This inhibition leads to the downregulation of key glycolytic enzymes (GLUT1 and LDHA), consequently reducing macrophage glycolysis and pro-inflammatory cytokine production, highlighting a metabolic mechanism underlying ASSD’s therapeutic action in RA.

Ermiao San (EMS), a classic TCM formula, has also demonstrated effective efficacy in alleviating RA symptoms and pathological progression. As indicated previously, EMS exerts its therapeutic effects by suppressing the PI3K/AKT/mTOR signaling axis, subsequently downregulating HIF-1α expression (115). This inhibition leads to a marked reduction in key glycolytic enzymes (including HK2 and GLUT1), thereby attenuating the pathological glycolysis in RA-FLS and inhibiting their migration and invasion. Furthermore, another TCM prescription Qingre Huoxue Decoction (QRHXD) also demonstrates potent clinical efficacy in alleviating RA symptoms and reducing disease activity. According to integrated multi-omics analysis and animal experimental results, QRHXD exerts therapeutic effects by activating the AMPK signaling pathway and concurrently inhibiting the gluconeogenic enzyme FBP1 (116). This dual action is proposed to rewire cellular metabolism, potentially shifting the balance away from glycolysis/gluconeogenesis, thereby mitigating inflammatory response and joint destruction in RA.

### 5.2 TCM treats OA through the glycolysis pathway

#### 5.2.1 TCM monomers

##### 5.2.1.1 TCM inhibits OA cartilage metabolism through the glycolysis pathway

ICA is a bioactive flavonoid derived from Herba Epimedii, exhibiting chondroprotective effects through its anti-inflammatory and antioxidant properties. As revealed by Wang et al., ICA could enhance chondrocyte viability by critically promoting anaerobic glycolysis (117). The treatment upregulates key glycolytic enzymes (including GLUT1, G6PD, PGK1, and PDK1), facilitating glucose uptake and metabolic flux through this pathway. This glycolytic shift, potentially mediated by increased HIF-1α expression, provides essential energy and biosynthetic precursors to support extracellular matrix synthesis, highlighting a key mechanism underlying its potential therapeutic application in OA.

α-Solanine, a glycoalkaloid found in plants of the Solanaceae family, has garnered attention for its anti-proliferative and pro-apoptotic effects against various cancer cell lines. In contrast, ICA has been demonstrated to enhance chondrocyte vitality by potently promoting anaerobic glycolysis (118). It upregulates key glycolytic enzymes (including GLUT1, G6PD, PGK1, and PDK1), facilitating glucose uptake, metabolic flux, and inhibition of ferroptosis. These findings suggest that α-Solanine inhibits the intense glycolysis associated with OA via the HIF-1α pathway and alleviates ferroptosis in chondrocytes, thereby offering a new therapeutic strategy for OA.

Harpagide, a natural iridoid glycoside, exhibits significant anti-inflammatory and chondroprotective properties. It has been demonstrated to mitigate TNF-α-induced inflammatory response in chondrocytes by modulating glycolytic metabolism (119). Specifically, harpagide restores the expression of key glycolytic enzymes (such as HK2, PFKP, and PKM), thereby regulating the glycolytic pathway. This metabolic reprogramming subsequently inhibits the production of pro-inflammatory mediators (such as IL-6 and COX-2), highlighting its potential as a therapeutic agent for OA via glycolysis targeting.

Songorine, a natural diterpenoid alkaloid, exhibits potent anti-inflammatory and chondroprotective effects by acting as a metabolic modulator. It exerts its therapeutic action in OA by directly targeting the glycolytic enzyme PFKFB3 (120). This binding inhibits glycolytic flux and lactate production, thereby disrupting the pathogenic positive feedback loop between glycolysis and inflammation. The consequent reduction in histone H4K12 lactylation downregulates pro-inflammatory gene expression, positioning songorine as a promising metabolic reprogramming agent for OA therapy.

##### 5.2.1.2 TCM inhibits OA macrophages through the glycolysis pathway

TCM can modulate macrophage polarization and metabolic reprogramming to alleviate inflammation in OA. As evidenced recently, songorine notably inhibits glycolysis in macrophages, demonstrated by reduced extracellular acidification rate and downregulated glycolytic genes (121). This metabolic shift promotes mitochondrial oxidative phosphorylation, decreases oxidative stress, and suppresses pro-inflammatory responses, ultimately mitigating OA progression through reprogramming of cellular energy metabolism.

##### 5.2.1.3 TCM compounds target glycolysis in OA

Tougu Xiaotong capsules (TGXTC) are a herbal compound preparation containing Morindae officinalis, Paeonia lactiflora Pall, Ligusticum wallichii, and Sarcandra glabra (License number in Fujian Province of Food and Drug Administration: MINZHIZI Z20100006) and are widely used to treat OA in China. A recent study has indicated that TGXTC ameliorates glycolytic metabolism disorder in OA chondrocytes by upregulating circFOXO3 (122). Both *in-vivo* and *in-vitro* experiments revealed that TGXTC notably suppressed the expression of key glycolytic enzymes and transporters (including GLUT1, HK2, PKM2, and LDHA), along with MMP13. Silencing circFOXO3 attenuated these regulatory effects, underscoring the critical role of circFOXO3 in mediating TGXTC’s action on glycolysis.

### 5.3 TCM treats SLE through the glycolysis pathway

The TCM formulation Qihuang Jianpi Zishen Granule (QJZG) is a compounded preparation comprising Astragalus membranaceus (Huangqi), Cuscuta chinensis Lam. (Yantuan Si), Rehmannia glutinosa Libosch. (Shu Dihuang), Dioscorea opposita Thunb. (Shanyao), Atractylodes macrocephala Koidz. (Fuchaobaizhu), Poria cocos (Fuling), Rubus chingii Hu (Fupenzi), and Rosa laevigata Michx. (Jinyingzi). This formula is developed and standardized by the First Affiliated Hospital of Anhui University of Chinese Medicine. Recently, Qian et al. have confirmed that QJZG ameliorates renal injury in lupus mice by modulating glycolytic metabolism (123). The results demonstrate that QJZG notably downregulates the expression of key glycolytic enzymes (HK2 and GLUT1), concurrently activating the AMPK/ULK1 pathway. This suppression of glycolysis is critically associated with inhibited M1 macrophage polarization, highlighting a potential mechanism through which QJZG attenuates inflammation and protects renal function.

The Soufeng Sanjie formula (SF) is composed of Astragali Radix (dried root of Astragalus membranaceus (Fisch.) Bge), scorpion (dried body of Buthus martensii Karsch), scolopendra (dried body of Scolopendra subspinipes mutilans L. Koch), and black soybean seed coats (seed coats of Glycine max (L.) Merr). It is an in-hospital preparation used for the treatment of rheumatoid immune diseases, which has been used clinically for many years with remarkable therapeutic effect. SF alleviates lupus and associated joint injury by targeting glycolytic metabolism in monocytic myeloid-derived suppressor cells (M-MDSCs). According to Tan et al., SF, via its active component delphinidin chloride, downregulates ALKBH5-mediated m6A modification to enhance FoxO1 expression (124). FoxO1 subsequently suppresses PFKFB3 transcription, thereby inhibiting glycolysis in M-MDSCs, enhancing their immunosuppressive function, and ultimately attenuating Th17 cell-mediated pathology. This study identifies that SF exerts its immunomodulatory effects in SLE primarily by suppressing glycolysis in monocytic M-MDSCs through the ALKBH5-FoxO1-PFKFB3 axis.

Jieduquyuziyin prescription (JP) is composed of 10 herbs: Rehmannia glutinosa (Gaert.), Trionyx sinensis Wiegmann, Artemisia annua L., Scleromitrion diffusum (Willd.) R.J.Wang (syn. Hedyotis diffusa Willd.), Paeonia anomalasubsp. veitchii (Lynch) D. Y. Hong, and K. Y. Pan (syn. Paeonia veitchii Lynch), Centella asiatica (L.), Actaea cimicifuga L. (syn. Cimicifuga foetida L.). JP therapy has demonstrated notable clinical benefits in ameliorating symptoms and reducing disease activity in SLE patients. As indicated by Li et al., JP alleviates SLE by targeting B cell metabolic reprogramming (125). Specifically, JP suppresses glycolysis in activated B cells through the AMPK/PKM2 signaling pathway, leading to reduced levels of glycolytic metabolites and enzymes, thereby inhibiting B cell hyperactivation and improving disease pathology. Similarly, Zhu et al. have also demonstrated that JP ameliorates SLE by modulating T cell metabolism (126). JP inhibits glycolysis in CD4^+^ T cells via the AMPK/mTOR pathway, as evidenced by decreased levels of glycolytic intermediates and key enzymes. This metabolic shift promotes Treg differentiation while suppressing Th17 cells, thereby restoring immune homeostasis in SLE models. Taken together, these studies highlight that JP exerts therapeutic effects in SLE primarily by targeting glycolytic pathways, suppressing glycolysis in B cells through AMPK/PKM2 and in T cells via AMPK/mTOR, thereby rebalancing immune responses and offering a metabolic intervention strategy for autoimmune diseases.

## 6 Challenges and future perspectives

Despite significant advances in elucidating the role of glycolytic reprogramming in ARDs, several limitations persist. Many studies remain at the preclinical stage, relying heavily on animal models or *in-vitro* systems that may not fully recapitulate the complexity of human immunometabolism. The heterogeneity among ARD subtypes and individual patients further complicates the generalization of findings. Moreover, the causal relationship between metabolic alterations and disease pathogenesis is not always clearly established, and off-target effects of metabolic inhibitors raise concerns regarding their clinical applicability. Additionally, while TCM shows promise in modulating glycolysis, the active components, precise targets, and underlying mechanisms of many TCM formulations remain incompletely characterized, limiting their standardization and widespread adoption.

Glycolytic enzymes and intermediates (such as PKM2, HIF-1α, LDHA, and lactate) have emerged as potential biomarkers for disease activity and progression in ARDs. The integration of metabolomic, proteomic, and transcriptomic data offers a powerful approach to identify novel metabolic signatures associated with specific ARD phenotypes. For instance, circulating levels of tumor M2-PK in RA or glycolytic intermediates in SLE may serve as non-invasive indicators of disease severity or treatment response. Future studies should prioritize the validation of these biomarkers in large, multi-center cohorts to establish their diagnostic and prognostic utility.

The heterogeneity in metabolic profiles among ARD patients underscores the critical need for personalized therapeutic strategies. Individual variations in genetic background, immune cell subsets, and metabolic enzyme expression may significantly influence responses to glycolytic inhibitors or TCM interventions. Personalized immunometabolic therapy aims to tailor treatments based on an individual’s distinct metabolic phenotype, potentially improving efficacy and minimizing adverse effects. With its multi-target and holistic approach, TCM is well-positioned to contribute to this paradigm, especially when combined with modern diagnostics to identify patient-specific metabolic dysregulations.

The integration of multi-omics technologies (including genomics, transcriptomics, proteomics, metabolomics, and epigenomics), provides a comprehensive framework to decode the complex metabolic networks underlying ARDs. Artificial intelligence (AI) and machine learning algorithms can analyze these high-dimensional datasets to identify key regulatory nodes, predict patient subgroups, and discover novel therapeutic targets. For example, AI-driven models have already been employed to identify glycolysis-related genes in OA with high diagnostic accuracy. Future research should leverage these advanced tools to map dynamic metabolic changes during disease progression and in response to TCM treatment, paving the way for precision medicine in ARDs.

## 7 Conclusion

In conclusion, accumulating evidence indicates that glycolytic reprogramming represents a central immunometabolic mechanism underlying the pathogenesis of ARDs. Across RA, OA, and SLE, enhanced aerobic glycolysis supports the activation and pathogenic functions of multiple immune and stromal cell populations, including T cells, B cells, macrophages, synovial fibroblasts, and chondrocytes. By regulating cellular bioenergetics, biosynthetic pathways, and inflammatory signaling networks, glycolytic metabolism contributes to immune dysregulation, chronic inflammation, and progressive tissue damage in ARDs.

Importantly, this review highlights several key regulatory nodes involved in glycolytic reprogramming, including HIF-1α, PKM2, PFKFB3, mTOR signaling, and lactate-mediated epigenetic modifications. These metabolic regulators form interconnected networks that integrate immune signaling with metabolic adaptation, thereby amplifying inflammatory responses across different cellular compartments.

Therapeutically, targeting glycolytic pathways has emerged as a promising strategy for ARD treatment. Both conventional metabolic inhibitors and bioactive compounds derived fromTCM have shown potential to suppress pathogenic glycolysis, restore immune metabolic balance, and alleviate disease progression in experimental models. Owing to its multi-component and multi-target characteristics, TCM provides a unique systems-level approach for modulating immunometabolic pathways.

Nevertheless, several challenges remain. Most current findings are derived from preclinical studies, and the clinical translation of glycolysis-targeted therapies requires further validation. Future studies should integrate multi-omics technologies, systems biology approaches, and artificial intelligence-based analyses to better characterize metabolic heterogeneity among ARD patients, identify reliable metabolic biomarkers, and develop personalized immunometabolic therapeutic strategies. A deeper mechanistic understanding of how TCM regulates glycolytic pathways may further facilitate the development of novel metabolic immunomodulators for ARDs.

Overall, elucidating the interplay between immune regulation and metabolic reprogramming will be essential for advancing both the mechanistic understanding and therapeutic management of autoimmune rheumatic diseases.
